# The Ketogenic Effect of SGLT-2 Inhibitors—Beneficial or Harmful?

**DOI:** 10.3390/jcdd10110465

**Published:** 2023-11-16

**Authors:** Michail Koutentakis, Jakub Kuciński, Damian Świeczkowski, Stanisław Surma, Krzysztof J. Filipiak, Aleksandra Gąsecka

**Affiliations:** 1 1st Chair and Department of Cardiology, Medical University of Warsaw, Banacha 1A, 02-097 Warsaw, Poland; mkoutentakis6@gmail.com; 2 Central Clinical Hospital, Medical University of Warsaw, Banacha 1A, 02-097 Warsaw, Poland; kuba.kucinski.5@gmail.com; 3 Department of Toxicology, Faculty of Pharmacy, Medical University of Gdansk, 80-416 Gdańsk, Poland; d.swieczkowski@gumed.edu.pl; 4 Faculty of Medical Sciences in Katowice, Medical University of Silesia, 40-752 Katowice, Poland; stasiu.surma@onet.eu; 5 Department of Clinical Sciences, Maria Sklodowska-Curie Medical Academy, 00-001 Warsaw, Poland; krzysztof.filipiak@uczelniamedyczna.com.pl; 6 Department of Hypertensiology, Angiology and Internal Medicine, Poznań University of Medical Sciences, 61-848 Poznań, Poland

**Keywords:** SGLT-2 inhibitors (gliflozins), T2DM, cardiovascular, ketogenesis, benefits, risks, ketogenic diet, infections, kidney disease, heart failure

## Abstract

Sodium–glucose cotransporter-2 (SGLT-2) inhibitors, also called gliflozins or flozins, are a class of drugs that have been increasingly used in the management of type 2 diabetes mellitus (T2DM) due to their glucose-lowering, cardiovascular (CV), and renal positive effects. However, recent studies suggest that SGLT-2 inhibitors might also have a ketogenic effect, increasing ketone body production. While this can be beneficial for some patients, it may also result in several potential unfavorable effects, such as decreased bone mineral density, infections, and ketoacidosis, among others. Due to the intricate and multifaceted impact caused by SGLT-2 inhibitors, this initially anti-diabetic class of medications has been effectively used to treat both patients with chronic kidney disease (CKD) and those with heart failure (HF). Additionally, their therapeutic potential appears to extend beyond the currently investigated conditions. The objective of this review article is to present a thorough summary of the latest research on the mechanism of action of SGLT-2 inhibitors, their ketogenesis, and their potential synergy with the ketogenic diet for managing diabetes. The article particularly discusses the benefits and risks of combining SGLT-2 inhibitors with the ketogenic diet and their clinical applications and compares them with other anti-diabetic agents in terms of ketogenic effects. It also explores future directions regarding the ketogenic effects of SGLT-2 inhibitors.

## 1. Introduction

SGLT-2 (sodium–glucose cotransporter-2) inhibitors, often referred to as gliflozins or flozins, achieved a significant milestone in 2012 with approval by the European Medicines Agency (EMA) and the Food and Drug Administration (FDA) for the treatment of type 2 diabetes mellitus (T2DM) [1,2,3]. This moment marked a paradigm shift in addressing elevated blood glucose levels. Within this context, the triad of endorsed SGLT-2 inhibitors, dapagliflozin (DAPA), canagliflozin (CANA), and empagliflozin (EMPA), emerged as pivotal components in the management of hyperglycemia. By concurrently modulating renal glucose reabsorption and enhancing urinary glucose excretion, this trio presented a potent therapeutic avenue for ameliorating the impact of T2DM [4,5,6,7]. Additional information on the recommended dose, indications, beneficial effects, and side effects of the above flozins is presented in Table 1.

While initially pursued for cardiovascular safety validation, SGLT2 inhibitors defied expectations in cardiovascular outcome trials (CVOTs) like EMPA-REG OUTCOME (Empagliflozin Cardiovascular Outcome Event Trial in T2DM Patients), CANVAS (Canagliflozin Cardiovascular Assessment Study), DECLARE-TIMI-58 (Dapagliflozin Effect on Cardiovascular Events−Thrombolysis In MI (Myocardial Infarction) 58), VERTIS CV (Evaluation of Ertugliflozin Efficacy and Safety Cardiovascular outcomes), and SCORED (Effect of Sotagliflozin on Cardiovascular and Renal Events in Patients with T2DM and Moderate Renal Impairment Who Are at Cardiovascular Risk). These trials surprisingly unveiled SGLT2 inhibitors’ ability to significantly reduce major adverse cardiovascular events (MACEs) compared to placebos. Notably, the 2015 EMPA-REG OUTCOME trial first demonstrated this protective effect, showcasing a 14% MACE reduction, 34% lower all-cause mortality, and 35% fewer heart failure (HF) hospitalizations [8]. Subsequent studies, DAPA-HF (Dapagliflozin and Prevention of Adverse Outcomes in HF) and EMPEROR-Reduced (Empagliflozin Outcome Trial in Patients with Chronic HFrEF (Heart Failure with Reduced Ejection Fraction)), revealed SGLT2 inhibitors’ transformative potential in HF treatment for patients with a reduced EF (ejection fraction) <40%, regardless of T2DM, decreasing hospitalizations, and mortality [9,10]. The EMPEROR-Preserved trial (Empagliflozin Outcome Trial in Patients with Chronic HFpEF (Heart Failure with Preserved Ejection Fraction)) in 2021 extended these benefits to chronic HF patients, irrespective of EFs, emphasizing SGLT2 inhibitors’ expanding role in enhancing prognosis [11].

In addition to that, the discovery of gliflozins’ nephroprotective effect has greatly impacted clinical practice as well. Diabetes is associated with microvascular damage, often culminating in chronic kidney disease (CKD) for around 40% of patients. Investigated in CVOTs, gliflozins effectively mitigate declines in the glomerular filtration rate (GFR), delay microalbuminuria, and hinder proteinuria progression, favoring both diabetic and nondiabetic patients. Recent EMPA-KIDNEY (The Study of Heart and Kidney Protection With Empagliflozin) trial data show SGLT2 inhibitors’ efficacy in nephropathy, even with a diminished eGFR (estimated glomerular filtration rate). Consequently, SGLT2 inhibitors assume a pivotal role in reducing the progression to end-stage renal disease (ESRD) among patients with CKD [12].

Beyond the aforementioned effects, SGLT-2 inhibitors have a pro-ketogenic effect that has been associated with their potential to increase the production of ketone bodies, such as BHB (β-hydroxybutyrate) [13]. This pro-ketogenic effect holds promising implications for cardiovascular events such as stroke and HF, as well as for retarding the progression of chronic diseases, such as atherosclerosis and CKD [14,15].

Research highlights that the ketogenesis triggered by SGLT-2 inhibitors contributes to anti-oxidative effects and the preservation of mitochondrial integrity in human disease models [16]. Nevertheless, there was also evidence that the use of these drugs may increase the risk of ketoacidosis in patients with T1DM (type 1 diabetes mellitus), so their further studies on T1DM were abandoned [17,18]. For years, diabetes specialists were conservative in their assessment of the potential benefits of ketogenesis, in contrast to cardiologists, who emphasized the benefits of ketone bodies for heart metabolism. As a result, the ketogenic effect of SGLT2 inhibitors has only been discussed recently. Ongoing clinical trials are still actively investigating the efficacy and safety of SGLT2 inhibitors for diabetes management and its associated complications [19]. Nonetheless, achieving a comprehensive understanding of their potential benefits and risks across diverse patient populations requires further research [20]. To what extent the ketogenic effect of these medications holds therapeutic potential for various conditions must be determined, even if some declare potential risks [21].

To our knowledge, this review article presents a thorough summary of the latest research on the mechanism of action of SGLT-2 inhibitors, their ketogenesis, and their potential synergy with the ketogenic diet for managing diabetes. The article particularly discusses the benefits and risks of combining SGLT-2 inhibitors with the ketogenic diet and their clinical applications, and compares them with other anti-diabetic agents in terms of their ketogenic effects. It also explores future directions with respect to the ketogenic effects of SGLT-2 inhibitors.

**Table 1:** Summary of the approved doses, indications, benefits, and adverse effects of the most commonly used SGLT-2 inhibitors.

Generic Name (Brand Name)	Dose (mg) Based on eGFR	Indications	Beneficial Effects	Adverse Effects
Canagliflozin (Invokana) [22]	eGFR > 60: 100 mg once daily and if tolerated, may increase to 300 mg once daily for additional glycemic control eGFR 30–60: 100 mg once daily eGFR < 30: initiation is not recommended, although patients with albuminuria (>300 mg/day) may continue 100 mg once daily to reduce the risk of ESRD, CV death, and hospitalization for HF On dialysis: contraindicated	Adjunct to diet and exercise Glycemic control in T2DM Heart attacks, stroke DKD CKD	↓Blood sugar levels ↓Body weight ↓BP ↓Risk of CV events in high-risk patients with T2DM ↓Risk of kidney disease progression	↑Risk of mycotic yeast infections, UTIs ↑Dehydration ↑Hypotension ↑Ketoacidosis ↑Risk of fractures ↑Risk of amputations in patients with PAD ↑AKI
Dapagliflozin (Farxiga) [23]	eGFR > 45: (a) initiation with 5 mg once daily if for glycemic control only. If tolerated, may increase to 10 mg once daily for additional glycemic control. (b) if other indications: initiation with 10 mg once daily eGFR 25–45: 10 mg once daily eGFR < 25: initiation is not recommended, but patients may continue 10 mg once daily to reduce the risk of eGFR decline, ESRD, CV death and hospitalization for HF On dialysis: contraindicated	Adjunct to diet and exercise Glycemic control in T2DM Chronic HFrEF ,CKD	↓Blood sugar levels ↓Body weight ↓BP ↓Risk of CV events in high-risk patients with T2DM ↓Risk of kidney disease progression ↓Risk of HF hospitalizations ↓Reduces MAFLD ↑Improves long-term glycemic control	↑Risk of mycotic yeast infections, UTIs ↑Dehydration ↑Hypotension ↑Ketoacidosis ↑Risk of fractures ↑Risk of amputations in patients with PAD ↑AKI
Empagliflozin (Jardiance) [24]	eGFR ≥ 30: initiation with 10 mg once daily. If tolerated, may increase to 25 mg once daily for additional glycemic control eGFR < 30: patients with T2DM and CV disease: no specific recommendations (lack of substantial evidence) eGFR < 20: patients with HF: no specific recommendations (lack of substantial evidence) On dialysis: contraindicated	Adjunct to diet and exercise Glycemic control in T2DM Chronic HFrEF CKD	↓Blood sugar levels ↓Body weight ↓BP ↓Risk of HF hospitalizations ↓CV mortality ↓Albuminuria ↑Improves arterial stiffness and endothelial function	↑Risk of mycotic yeast infections, UTIs ↑Dehydration ↑Hypotension ↑Ketoacidosis ↑Risk of fractures ↑Risk of hyperkalemia ↑AKI
Ertugliflozin (Steglatro) [25]	eGFR ≥ 45: (a) initiation with 5 mg once daily. If tolerated, may increase to 15 mg once daily for additional glycemic control eGFR < 45: it is not recommended On dialysis: contraindicated	Adjunct to diet and exercise Glycemic control in T2DM	↓Blood sugar levels ↓Body weight ↓BP	↑Risk of mycotic yeast infections, UTIs ↑Dehydration ↑Ketoacidosis

Abbreviations: ↓—decrease; ↑—increase; eGFR—estimated glomerular filtration rate; ESRD—end-stage renal disease; BP—blood pressure; CV—cardiovascular; T2DM—type 2 diabetes mellitus; DKD—diabetic kidney disease; CKD—chronic kidney disease; UTIs—urinary tract infections; PAD—peripheral artery disease; AKI—acute kidney injury; HF—heart failure; MAFLD—metabolic-associated fatty liver disease; HFrEF—heart failure with reduced ejection fraction.

## 2. Mechanism of Action of SGLT-2 Inhibitors

Sodium–glucose cotransporters (SGLTs) are a class of transmembrane proteins that share a common transport mechanism in which extracellular sodium binding causes a gate to open and traps glucose from outside of the cell. The transporter then shifts, releasing sodium and glucose into the cytoplasm. The protein reverts to its initial conformation at the completion of the process. The two most common SGLTs are SGLT-1 (sodium–glucose cotransporter-1) and SGLT-2 (sodium–glucose cotransporter-2) [26,27].

The SGLT2 transporter is predominantly expressed in the epithelial cells of the renal proximal convoluted tubule. Although SGLT2 has a low affinity for glucose, it demonstrates a remarkable capacity for renal glucose reabsorption [28]. The process is primarily sodium-dependent, with SGLT2 and SGLT1 exhibiting ratios of 1:1 and 2:1, respectively. While SGLT1 is responsible for only approximately 10% of the tubular glucose reabsorption, SGLT2 handles the majority of the reabsorption. This capacity for reabsorbing filtered glucose in the kidneys is an extremely efficient energy conservation mechanism. Apart from the kidneys, SGLT2 expression has been detected in other organs such as the brain, liver, thyroid, muscles, and heart, while SGLT1 expression has been reported in the intestine, trachea, kidney, heart, brain, testes, and prostate. The high capacity of renal glucose reabsorption, especially by SGLT2 has led to the development of SGLT2 inhibitors as a treatment for T2DM [26].

Within this framework, SGLT-2 inhibitors function by obstructing the activity of the SGLT-2 protein, which is prominently present in the proximal convoluted tubules of the kidneys. This action effectively prevents the reabsorption of filtered glucose from the tubular lumen [29]. Their mechanism of action is based on the renal excretion of glucose, causing glucosuria, and is independent of insulin action, thus reducing hypoglycemia, weight gain, and liver disease [30].

Furthermore, it is still unknown how inhibiting glucose reabsorption in the kidneys increases insulin sensitivity in peripheral tissues like muscle and fat, so the paradoxical link between inhibition of SGLT2 and increased insulin sensitivity has been a topic of research interest in recent years [31]. Some researchers hypothesized that changes in the gut microbiome or adjustments to the secretion of hormones such as glucagon and GLP-1 (glucagon-like peptide 1) could significantly contribute to this observed rise in insulin sensitivity [32].

**Figure 1:**
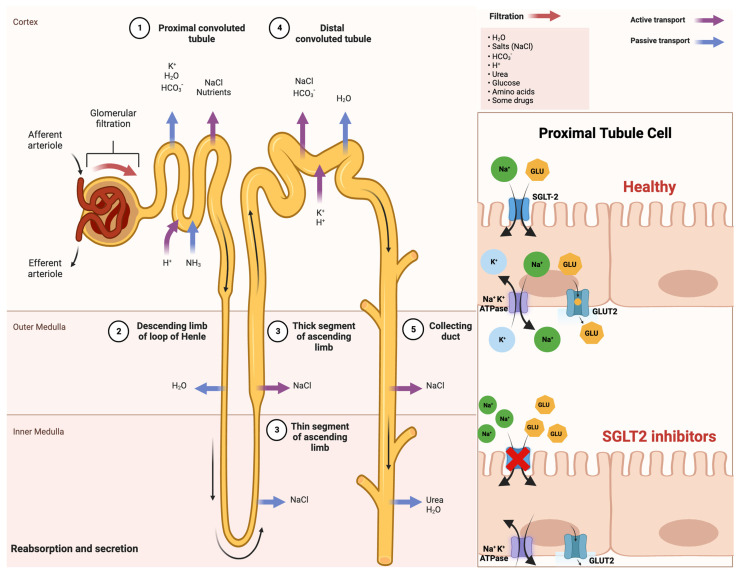
Comprehensive schematical depiction of the mechanism of action of SGLT2 inhibitors. Abbreviations: SGLT2—sodium−glucose cotransporter 2; Na^+^—sodium cations; K^+^—potassium cations; H_2_O—oxygen hydride (water); HCO_3_^−^—bicarbonate ions; NaCl—sodium chloride (salt); H^+^—hydrogen ions; NH_3_—ammonia; GLU—glucose; ATPase—adenosine triphosphatase (enzyme); GLUT2—glucose transporter 2. Created with BioRender.com and accessed on 17 April 2021.

An improved comprehension of SGLT2 inhibitors and their impact on insulin sensitivity holds promise for enhancing treatments for T2DM and metabolic disorders. This insight may also illuminate the intricate interplay between insulin sensitivity and glucose balance, which is vital for maintaining metabolic health. A schematic illustration that indicates the mechanism of action of SGLT-2 inhibitors in detail is shown in Figure 1.

As previously stated, SGLT-2 inhibitors have an influence not only on the kidney but also on the heart, liver, pancreas, and adipose tissue. Therefore, they are specially designed for T2DM, and they have been found to have additional benefits such as slowing the progression of kidney disease, reducing HFrEF [33], and lowering the risk of kidney failure and death in people with kidney disease and T2DM [34].

Although the exact mechanism of action of these drugs on the heart is still not completely understood, it is thought to be multifactorial. By lowering blood pressure and plasma volume, which may decrease preload and afterload, SGLT-2 inhibitors may aid in reducing the workload on the heart [35,36]. They may also have an anti-fibrotic impact, reducing the buildup of scar tissue and enhancing heart performance [37]. Furthermore, these drugs can reduce the risk of oxidative stress and enhance endothelial function, which may lessen the risk of cardiac muscle deterioration and enhance blood flow [37,38].

Even if SGLT-2 inhibitors have demonstrated efficacy in improving liver function, the exact mechanisms of action are currently unclear. These medications could potentially enhance liver function by reducing hepatic steatosis, inflammation, and oxidative stress [39,40,41]. Furthermore, flozins may increase the production of ketone bodies [42], which can serve as an alternative energy source for the liver and decrease hepatic glucose production. These benefits may lead to improvements in insulin sensitivity and liver glucose metabolism. Overall, SGLT-2 inhibitors have shown promise in the treatment of nonalcoholic steatohepatitis (NASH) [43] and metabolic-associated fatty liver disease (MAFLD) [40,43].

With respect to the effect of gliflozins on the pancreas, multiple studies have demonstrated that SGLT2 inhibitors enhance insulin secretion in T2DM patients [44]. The ratio of the incremental area under the plasma C-peptide concentration-versus-time curve to that under the plasma glucose concentration-versus-time curve during a 75 g oral glucose tolerance test was elevated substantially in T2DM patients receiving dapagliflozin compared with those receiving a placebo [45]. Patients with T2DM receiving empagliflozin also demonstrated improved pancreatic beta cell function, as measured by the insulin secretion/insulin resistance score during the hyperglycemic clamp technique [46].

While their main focus is not on adipose tissue, there is evidence to support the notion that SGLT-2 inhibitors may have an impact on fat metabolism [47]. According to some study findings, these inhibitors, especially in T2DM patients, may result in a small amount of weight loss [48,49] and a decrease in body fat, especially by decreasing TAG (triglyceride) accumulation [50]. Yet, more investigation is required to completely understand how SGLT-2 inhibitors affect adipose tissue and how this affects how fat is metabolized.

**Figure 2:**
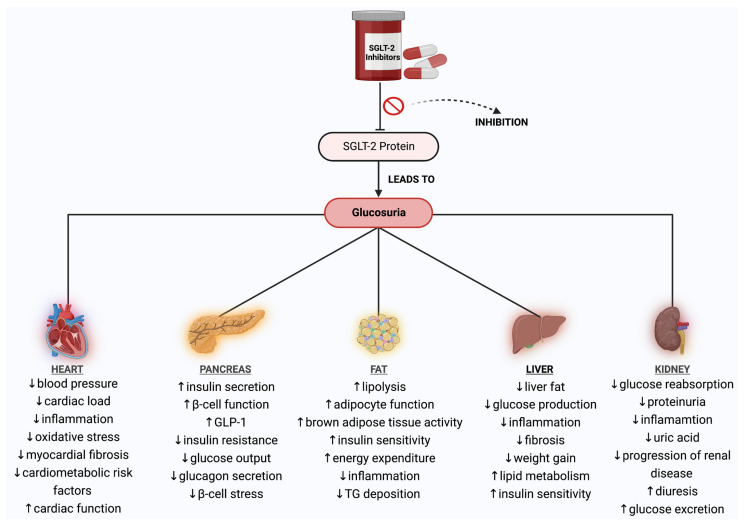
Schematic diagram of the effects and mechanisms of action of all SGLT-2 inhibitors on certain organs. Abbreviations: SGLT-2—sodium–glucose cotransporter-2; ↓—decrease; ↑—increase; GLP-1—glucagon−like peptide-1; TG—triglycerides. Created with BioRender.com and accessed on 17 April 2021.

Taking all the above into consideration, it is vital to understand that SGLT-2 inhibitors should only be used under the guidance of a healthcare professional and should not be used by individuals with T1DM or those with severe kidney disease, as their mechanism of action relies on adequate kidney function. Patients taking SGLT-2 inhibitors must also be monitored closely for possible side effects. The specific effects and underlying mechanisms of all SGLT-2 inhibitors on distinct organs are presented in Figure 2.

## 3. Synergism of SGLT-2 Inhibitors and Ketogenic Diet: Benefits and Risks

Type 2 diabetes (T2DM) is a global epidemic affecting millions of people worldwide [51,52]. More than 95% of people with diabetes have T2DM, which is mostly the result of excess body weight, according to the World Health Organization (WHO, 2011), and physical inactivity [53]. Recently, concerns have been raised that more than one-third of diabetes-related deaths occur in people under the age of 60 [54], and the burden of diabetes mellitus is rising at a much faster rate in developed regions, such as Western Europe [55]. These trends have been attributed to higher levels of unhealthy diet intake and sedentary behavior, which result in an elevated body mass index (BMI) and fasting plasma glucose [56]. In particular, people with a higher BMI are more likely to have T2DM [57].

In 2017, about 462 million individuals suffered from T2DM, corresponding to 6.28% of the world’s population [55]. By 2030, the global prevalence of T2DM is expected to increase to 7079 cases per 100,000 people, indicating an ongoing surge in all geographical areas. Therefore, the prevalence of this disease is increasing rapidly, and it is also estimated that by 2045, over 700 million people will be affected [58].

**Figure 3:**
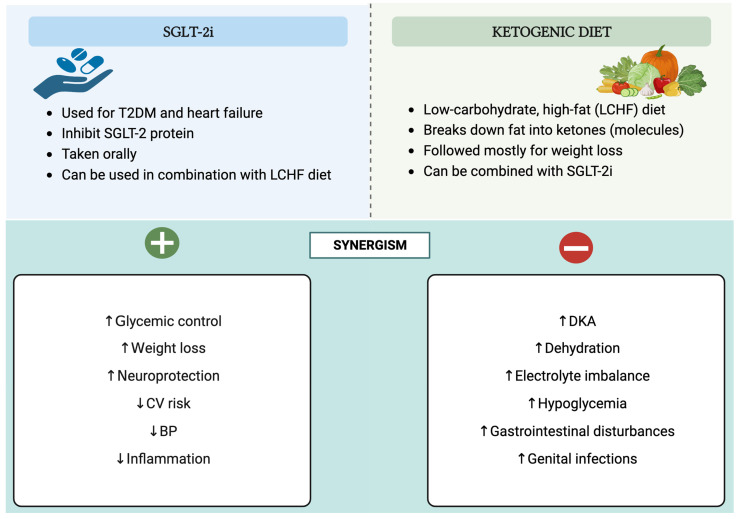
The attributes of SGLT-2 inhibitors and ketogenic diets, along with the potential benefits and risks of their combined influence. Abbreviations: SGLT-2i—sodium–glucose cotransporter-2 inhibitors; ↓—decrease; ↑—increase; T2DM—type 2 diabetes mellitus; LCHF—low-carbohydrate, high-fat; CV—cardiovascular; BP—blood pressure; DKA—diabetic ketoacidosis. Created with BioRender.com, accessed on 17 April 2021.

The management of T2DM is crucial to prevent complications and improve the quality of life of those affected. Having said that, one potential approach to managing T2DM is the synergistic use of SGLT-2 inhibitors and a ketogenic diet, which can offer benefits but also generate risks that need to be considered. A schematic and a tabular representation of the characteristics of SGLT-2 inhibitors and ketogenic diets, along with the potential strengths and limitations of their synergy, are shown in Figure 3 and Table 2, respectively.

### 3.1. Positive Synergistic Effect of SGLT-2 Inhibitors and Ketogenic Diet: Benefits

Ketogenic diets and SGLT2 inhibitors are two highly promising therapeutic approaches for treating T2DM and other complications. In recent years, there has been a noticeable increase in interest in both of these strategies due to their potential synergistic health merits, such as weight reduction, improved insulin sensitivity, and decreased cardiovascular risk [59,60,61].

As previously noted, SGLT-2 inhibitors are a group of oral anti-diabetic medications that prevent the kidneys from reabsorbing glucose, enhance urine glucose excretion, and lower blood sugar levels [62]. On the other hand, the ketogenic diet, characterized by its low-carbohydrate and high-fat composition [63,64], consisting approximately of 70–80% fat, 10–20% protein, and 5–10% carbohydrates, induces ketosis, a metabolic state in which the body primarily uses fat for energy production by producing ketones rather than glucose [65]. Both approaches independently promote weight loss and improve glycemic control, which are crucial for managing these conditions [66].

A notable aspect is that the development of mild ketosis in ketogenic diets has been hypothesized to contribute to the beneficial effects of SGLT2 inhibition on cardiac and renal outcomes [67]. Considering that patients find it challenging to maintain a low-carb, high-fat diet (LCHFD) for an extended period of time and that compliance is low, a mix of an LCHFD and SGLT-2 inhibitors may help to catabolize fat depots while causing less discomfort than a strict LCHFD alone. Such an experimental therapy would require tight clinical control.

In more detail, SGLT-2 inhibitors exert their weight loss effects through the increased urinary excretion of glucose, leading to a calorie deficit and a subsequent reduction in body weight. This weight loss is predominantly due to a decrease in adipose tissue, which is beneficial since excessive adipose tissue is primarily associated with insulin resistance and inflammation in T2DM [68]. A further correlation between SGLT-2 inhibitors and better glycemic control is the reduction in HbA1c (glycated hemoglobin) levels [69].

Similarly, the ketogenic diet has emerged as a promising dietary intervention for managing T2DM as it offers several advantages [70]. Firstly, the diet restricts the amount of carbohydrates consumed, lowering blood glucose levels and HbA1c levels, an indicator of long-term blood glucose management [71]. Secondly, as mentioned, the diet triggers a state of ketosis, which results in weight loss, especially in individuals with T2DM who are overweight or obese [66,72].

Notably, some studies explored the effectiveness of an LCHFD in promoting weight loss and reported greater weight losses in patients following this diet compared to a low-fat diet [73]. In addition, the LCHFD was found to be a safe nutritional intervention for managing obesity in terms of acid–base equilibrium [74]. Taken together, these results emphasize the potential of dietary interventions in the management of this chronic disease and highlight that a low-carbohydrate ketogenic diet (LCKD) is a feasible option for people wishing to manage both their weight and T2DM.

To amplify these therapeutic benefits, the combined use of SGLT-2 inhibitors with a ketogenic diet may prove to be an innovative tactic. In fact, recent studies have suggested that combining SGLT-2 inhibitors with ketogenic diets may have additive effects on glucose control and other metabolic parameters [75]. Taking into consideration the fact that both interventions can lead to a decrease in HbA1c [76,77], body weight [68,78], and blood pressure [79,80,81], it seems reasonable to say that the combination of the two would result in even greater reductions in HbA1c, body weight, and blood pressure than either treatment alone, thereby offering a more effective approach to managing T2DM and obesity. On top of that, considering the anti-inflammatory effects of both treatments [82,83], their simultaneous use may lower the risk of chronic inflammation and associated CVD, bringing about superior outcomes.

Numerous assertions have been put forth regarding the neuroprotective advantages attributed to both ketogenic diets and SGLT-2 inhibitors, further emphasizing their potential impact on neurological health [83,84,85]. While the only well-established use of a ketogenic diet is for reducing seizures in pediatric epilepsy [86], its ability to produce ketone bodies that serve as alternative fuels for brain metabolism is key to maintaining mitochondrial function, ATP (the source of energy for use and storage at cellular level) production, and neuronal survival. Likewise, there is mounting evidence that SGLT-2 inhibitors have neuroprotective properties [85,87]. Empagliflozin was reported to improve cerebral microvascular and cognitive impairment in a murine mixed model of diabetes mellitus and Alzheimer’s disease [88]. Other meta-analyses have shown that SGLT2 inhibitors may reduce the risk of hemorrhagic strokes by about 50% [89,90]. Therefore, the synergistic utilization of SGLT-2 inhibitors alongside a ketogenic diet holds the promise of yielding even more pronounced and noteworthy neuroprotective outcomes.

Despite these encouraging prospects, a more comprehensive evaluation of their potential positive synergistic effects is necessary before they can be widely implemented. While the individual therapeutic benefits of both SGLT-2 inhibitors and ketogenic diets have been extensively studied, the combined beneficial effects of these interventions are not largely known.

### 3.2. Negative Synergistic Effect of SGLT-2 Inhibitors and Ketogenic Diet: Risks

Very-low-carbohydrate diets, also known as ketogenic diets, were initially popular fad diets for weight reduction [91,92], but they are now used in medicine to treat diabetes, epilepsy, and obesity [91,92,93,94,95]. A rise in the concurrent use of ketogenic diets and SGLT-2 inhibitors to control weight in diabetic patients is due to the increased use of SGLT-2 inhibitors in the management of diabetes [28]. In spite of the potential synergistic benefits that might occur, the combination of these inhibitors and ketogenic diets may also pose certain risks [75].

Given that, one major concern is the increased risk of euglycemic diabetic ketoacidosis (euDKA, DKA), a potentially life-threatening emergency and potentially fatal condition characterized by the excessive production of ketone bodies, including BHB, and a subsequent decrease in blood pH [66,75,96]. DKA can be specifically distinguished by the triad of euglycemia (blood glucose less than 250 mg/dL) in the presence of severe metabolic acidosis (arterial pH less than 7.3, serum bicarbonate less than 18 mEq/L) and ketonemia [97]. It should be highlighted that euDKA can appear in patients with both T1DM and T2DM. Because euglycemic ketosis can be brought on by either a ketogenic diet or SGLT-2 inhibitors alone [21,98,99,100], there is probably a greater possibility that it will occur when both therapies are combined.

There have been cases of patients who developed life-threatening euDKA while adhering to a strict ketogenic diet with concomitant SGLT-2 inhibitor use. One of them described a 73-year-old female patient who used an SGLT2 inhibitor with adherence to a ketogenic diet, which contributed to a severe state of ketone production and euDKA in the perioperative period [101]. Another study documented two patients in which euDKA occurred after just one dose of an SGLT2 inhibitor while on a ketogenic diet [96].

Another potential risk is the possibility that the combination of SGLT-2 inhibitors and a ketogenic diet could result in dehydration [96]. SGLT-2 inhibitors, through their mechanism of action, may cause increased glucose excretion in the urine, along with an increased excretion of water and electrolytes, including sodium [81]. The ketogenic diet can also lead to increased water and electrolyte loss due to the diuretic effect of ketones [93]. As a result, combining these two therapies may increase the risk of both dehydration and electrolyte imbalances.

In this regard, the symptoms of dehydration caused by SGLT-2 inhibitors and following an LCKD can include increased thirst, dry mouth/tongue, dark yellow urine, fatigue, dizziness, lethargy, and confusion [102,103]. In severe cases, dehydration can cause low blood pressure, tachycardia, and syncope [103]. For that reason, it is important to monitor fluid and electrolyte levels closely and seek medical advice if any concerning symptoms occur.

Moreover, a ketogenic diet and SGLT-2 inhibitors may rarely raise the risk of hypoglycemia, particularly in diabetics who are taking insulin or other glucose-lowering drugs [96,104]. While the ketogenic diet can also lower blood glucose levels by limiting carbohydrate consumption [105], SGLT-2 inhibitors lower blood glucose levels by increasing glucose excretion in the urine [106]. In light of this, combining these two treatments may result in a greater reduction in blood glucose, which could eventually cause hypoglycemia. To prevent this from happening, it is crucial to routinely check blood glucose levels and adjust medication dosages as needed.

It has also been suggested that the combination of SGLT-2 inhibitors and a ketogenic diet may result in gastrointestinal (GI) disturbances [96]. Nevertheless, both treatments can cause GI side effects independently, such as nausea, vomiting, and diarrhea [30,107]. Consequently, when using SGLT-2 inhibitors and following a ketogenic diet, patients should be evaluated for any signs of GI symptoms.

Other adverse effects may include infections, such as urinary and genital infections. To date, there is no concrete proof that using SGLT-2 inhibitors and a ketogenic diet can cause infections. What is known is that SGLT-2 inhibitors have been associated with an increased risk of genital and urinary tract infections owing to their mechanism of action [108], which involves reducing glucose reabsorption in the kidneys and provoking glycosuria. This can create an environment in the urinary tract that is more conducive to bacterial growth. Similarly, even though the ketogenic diet has an anti-inflammatory impact [109], it might not be followed properly, and thus it can lead to micronutrient deficiencies, such as folate (vitamin B9), biotin (vitamin B7), vitamins A, E, D, selenium, choline, chromium, iodine, magnesium, and molybdenum [110], that can weaken the immune system and increase the risk of infections.

With respect to the other side events that a ketogenic diet may cause, it is noteworthy to focus on a study presented at the recent American College of Cardiology (ACC) Congress, which indicated that an LCHFD (or keto-like diet), similar to the ketogenic diet, doubles the risk of cardiovascular events [111].

The data from the UK Biobank, a large-scale prospective database with health information from over half a million people living in the United Kingdom who were followed for at least 10 years, were analyzed in this context as well. Upon enrollment in the Biobank, 70,684 participants completed a one-time self-reported 24 h diet questionnaire and, at the same time, had blood drawn to check their levels of cholesterol. The researchers identified 305 participants whose questionnaire responses indicated that their diet during the 24 h reporting period met the study’s definition of an LCHFD. These participants were matched by age and sex with 1220 individuals who reported eating a standard diet. The result was that the average age of the study population was 54 years, and 73 percent were women. The mean BMI was 27.7 for those on the LCHFD and 26.7 for those on the standard diet.

In comparison to participants who followed a standard diet, those who followed an LCHFD had considerably increased levels of both LDL (low-density lipoprotein) cholesterol and apoB (apolipoprotein B), which is the protein component found on LDLs and other atherogenic lipoprotein particles. Despite making adjustments for other heart disease risk factors, such as obesity, diabetes, high blood pressure, and smoking, people on an LCHFD were over twice as likely to experience several significant cardiovascular events, such as coronary atherosclerosis that required revascularization, MI, stroke, and PAD (peripheral artery disease). In total, 9.8% of participants on an LCHFD encountered a new cardiovascular event, whereas 4.3% of those following a standard diet faced the same outcome—showing that there was almost double the risk for those following the LCHFD.

Additionally, a longer-term follow-up study of people on an LCHFD (less than 215 g/day of carbohydrates) revealed a 32% increase in the all-cause mortality among the participants, a 36% risk of dying from cancer, and a 50% mortality due to CVDs. Because the study was observational, a causal link between diet and an elevated risk for serious cardiac events cannot be established. However, researchers stated that the results merited more investigation in prospectively planned studies, particularly since about one in five Americans claims to follow a low-carb, keto-like, or full ketogenic diet.

According to claims, while an LCHFD may offer benefits for certain patients under medical supervision, adopting such a diet without proper professional guidance can lead to severe health consequences. In general, the use of SGLT-2 inhibitors in conjunction with a strict LCHFD is considered to be contraindicated.

## 4. Clinical Applications of SGLT-2 Inhibitors as Ketogenic Agents

SGLT-2 inhibitors exhibit protective effects on the heart, liver, and kidneys. They specifically exert an anti-hyperlipidemic, anti-atherosclerotic, anti-obesity, and anti-neoplastic impact as per in vitro, pre-clinical, and clinical studies. The pleiotropic effects of this class have been attributed to a variety of pharmacodynamic actions such as natriuresis, hemoconcentration, inhibition of the renin–angiotensin–aldosterone system, alterations in energy homeostasis, glycosuria, lipolysis, anti-inflammatory and antioxidative actions, and ketone body formation (ketogenesis) [36,112].

SGLT-2 inhibitors have been subject to scrutiny with regard to their viability as ketogenic agents not only for the management of T2DM [113] but also for other conditions, including obesity [68], kidney disease, and HF [62], MAFLD [40], and metabolic syndrome [114]. Empirical evidence gleaned from studies conducted on both animal and human subjects indicates a consistent report of the ketogenic effect of these drugs [115,116,117,118,119,120,121].

### 4.1. Type 2 Diabetes Mellitus

Over the past two decades, the treatment of T2DM has undergone continuous refinement, representing an ever-evolving discipline within the medical sciences. The frequency of severe catastrophic effects, including amputations, renal failure requiring dialysis, and blindness from retinopathy, has considerably declined as a result of therapeutic advancements. Drug development has achieved therapeutic objectives. That being said, millions of people with T2DM may benefit from the glycemic and nonglycemic effects of the inhibitors of SGLT-2, a class of drugs with remarkable versatility.

The selection of pharmacotherapy for T2DM is largely dependent on the comorbidity of CVD and CKD. This is in accordance with the joint recommendations of the American Diabetes Association (ADA) and the European Association for the Study of Diabetes (EASD), initially published in 2018 and subsequently revised in late 2022 [122,123]. The guidelines established by the European Society of Cardiology (ESC) in 2023 also have a vital impact on influencing these decisions [124].

The current guidelines, developed by the SCORE2-Diabetes Working Group and the ESC Cardiovascular Risk Collaboration, provide evidence-based suggestions for managing cardiovascular risk in individuals with diabetes and offer guidance on how to tackle atherosclerotic cardiovascular disease (ASCVD) in patients with diabetes. The guidelines introduce a novel, 10-year cardiovascular risk assessment tool, SCORE2-Diabetes, for patients with T2DM who do not have ASCVD or significant target organ damage (TOD). This expanded version of the established SCORE2 prediction model for T2DM estimates the 10-year probability of fatal and nonfatal cardiovascular events (like an MI/heart attack or stroke) based on the patient’s individual characteristics. The SCORE2-Diabetes score is a useful tool for clinical decision making for patients with T2DM at different risk levels—low, moderate, high, or very high—but without clinically evident ASCVD or severe TOD [124,125].

Considering the notable incidence of undiagnosed diabetes in patients with CVD and the increased risk and treatment implications when both conditions are present these guidelines advise routine diabetes screening for all CVD patients. Furthermore, all diabetes patients should be assessed for the risk and presence of CVD and CKD. The current guidelines, based on extensive CVOTs, provide explicit instructions on how to manage patients with diabetes and clinical signs of cardiovascular-renal disease. Therefore, in patients with diabetes and ASCVD, GLP-1 receptor agonists and/or SGLT2 inhibitors are recommended to lower the cardiovascular risk, regardless of glucose control, in addition to standard care such as antiplatelet, antihypertensive, or lipid-lowering therapy. These guidelines emphasize managing HF in diabetes, an area that has been overlooked for years. Based on the data from CVOTs, it is advised to treat diabetes patients with chronic HF (regardless of LVEF) with SGLT2 inhibitors to decrease HF hospitalization. Lastly, in patients with diabetes and CKD, treatment with an SGLT2 inhibitor and/or finerenone is recommended, as these agents reduce cardiovascular and kidney failure risk in addition to standard care [124].

Managing coexisting conditions like T2DM and CVD necessitates a multidisciplinary approach. The collective expertise of different healthcare professionals is crucial in making informed decisions and implementing individualized treatment approaches with the goal of decreasing the impact of the patient’s health condition. The ultimate goal of managing CVD in diabetic patients is not only to improve their prognoses but also to enhance their overall quality of health and life. It is crucial to strictly follow guidelines, currently emphasizing the need to address HF and promoting the use of certain drugs like GLP-1 receptor agonists and SGLT2 inhibitors. With the help of tools like SCORE2-Diabetes, clinical decisions are adjusted to individual T2DM patients’ risk levels, adding to the effectiveness of the personalized care approach.

### 4.2. Obesity

In the last 15 years, the prevalence of obesity has doubled, and it is becoming a growing health problem that has gained pandemic dimensions. Based on data from the World Health Organization, in 2016, roughly 40% of the global adult population, equating to 1.9 billion individuals, were affected by overweight or obesity, with 650 million among them exhibiting a more severe form of obesity [126].

In addition to the cornerstone of overweight and obesity, which is lifestyle modification, adjuvant pharmacotherapies, such as SGLT2 inhibitors, instantly reduce body weight by causing the kidneys to excrete glucose, which results in calorie loss. Amounts of 60–100 g of glucose per day may be eliminated in the urine as a result of SGLT2 antagonism, which functions in a glucose-dependent way [68].

In this context, numerous studies have consistently demonstrated the weight loss efficacy associated with SGLT2 inhibitor therapy in patients with T2DM, regardless of whether these individuals are administered SGLT2 inhibitors alone or in conjunction with other glucose-lowering therapies [68].

In compliance with findings from certain meta-analyses, all SGLT2 inhibitor treatments have demonstrated reductions in body weight of approximately 1.5–2 kg compared to a placebo [127,128,129,130], with the magnitude of this effect being dose-dependent [131]. Clinical evidence spanning up to four years indicates that the capacity of these drugs to elicit reductions in body weight is sustained over the course of treatment [132,133,134].

Interestingly, only a few studies have examined how SGLT2 inhibitors affect weight reduction in obese people without diabetes. In those studies, canagliflozin 100 mg alone was shown to lower body weight by 2.8 kg [135].

Moreover, in experiments involving diet-induced obese rats, it was discovered that the use of SGLT2 inhibitors caused elevated amounts of lipolysis and circulating ketone bodies [115,116]. In clinical trials involving patients diagnosed with T2DM or obesity without diabetes, SGLT2 inhibitor-induced glycosuria led to a decrease in plasma glucose and insulin levels while simultaneously increasing both fasting and post-meal glucagon concentrations. The resulting fall in glucose concentration and the associated hormonal changes encouraged the mobilization of lipid storage [136], which ultimately resulted in changes in energy substrate utilization that favored the use of lipids for energy production [118].

Finally, in other animal models, SGLT2 inhibitors were found to decrease adipose tissue inflammation and enhance brown adipose tissue [137,138]. Since low-grade persistent inflammation in adipose tissue is a key mediator in the development of obesity-associated conditions like insulin resistance and T2DM, reducing inflammation in adipose tissue would be of particular importance to obese people [139].

### 4.3. Heart Failure

Heart failure (HF) affects approximately 40 million individuals globally. The prevalence of HF is escalating to epidemic proportions, which can be attributed in part to the worldwide burden of cardiovascular risk factors and the aging of populations [140,141,142,143,144]. Despite substantial progress made in medical and device-based therapies for HF patients [145] in recent decades, mortality rates remain alarmingly high [146].

Pharmacotherapy continues to be the foundation of HF treatment, and various potent novel therapeutic avenues have recently emerged [147]. These include angiotensin-neprilysin receptor inhibitors (ARNi) [148], SGLT-2 inhibitors, omecamtiv mecarbil (INN) [149], and vericiguat [150]. Among these options, SGLT-2 inhibitors, as ketogenic agents, have arguably demonstrated the most remarkable and consistent benefits across the HF spectrum, coupled with an outstanding safety profile [151].

It has been hypothesized that the oxidation of ketone bodies serves as an additional fuel source, which is highly energy-efficient [152] and efficiently extracted by the heart [9,10,153]. Furthermore, patients without T2DM with HFrEF who received a continuous infusion of BHB showed increases in CO (cardiac output), LVEF (left ventricular ejection fraction), and myocardial oxygen consumption without altering the myocardial external energy efficiency [154]. Additionally, animal models of MI that were subjected to treatment with empagliflozin or a ketone diet demonstrated elevated levels of circulating ketone bodies, resulting in improved left ventricular function [155,156,157].

It is reasonable to postulate that the low level of ketonemia caused by SGLT2 inhibitors could improve myocardial energy and contractile function. Elevating ketone body levels is a promising strategy for treating metabolic dysfunction in HF [158,159,160,161]. Recent results from the EMPA-TROPISM (Are the “Cardiac Benefits” of Empagliflozin Independent of Its Hypoglycemic Activity?) study showed that empagliflozin treatment improved left ventricular systolic function in HFrEF patients without T2DM [155]. To what degree ketone bodies affect energy metabolism and cardiac contractility, however, would require more investigation.

Until now, multiple studies have shown that SGLT2 inhibitors reduce HF-related mortality [154,162,163]. Increased ketogenesis as a result of SGLT-2 inhibitors may significantly change energy metabolism in cardiomyocytes (or cardiac cells), reducing energy deficits in the myocardium [164,165]. In HF, cardiomyocytes have a lower ability to use glucose or fatty acids. Therefore, ketones have become a more important fuel source, providing energy in a highly efficient manner with significant uptake by myocytes [165]. A heart deprived of oxygen can benefit from ketone body oxidation, as, when combined with fatty acids, they are more efficient in terms of oxygen utilization [165,166]. This mechanism has the potential to serve as an efficacious therapeutic strategy for addressing the metabolic aspect of HF by ameliorating O2 consumption and curtailing reactive oxygen species (ROS) production through the use of SGLT2 inhibitors. As a result, it could lead to more effective cardiac functioning and impede the progression of HF [154,162,165].

In addition to all its metabolic benefits, SGLT2 inhibitors seem to reverse cardiac remodeling, resulting in better myocardial function [165,167]. In detail, clinical trials such as DAPA-HF, EMPEROR-Reduced, and EMPA-TROPISM have consistently shown that SGLT2 inhibitors reverse and improve adverse cardiac remodeling in patients with HFrEF and HFpEF [11,168,169,170], which could explain the observed cardiovascular benefits [8,9]. Nevertheless, given that SGLT2 receptors are not typically expressed in cardiomyocytes [171], it is probable that the impact of SGLT2 inhibitors on the left ventricle is an indirect outcome, brought about by the hemodynamic, anti-inflammatory, and metabolic effects that they mediate [158,168,172].

The management of HFpEF has been a fundamental clinical need since the results of the EMPEROR-Preserved trial were published. This trial demonstrated for the first time a 21% risk reduction in the main composite outcome of cardiovascular mortality or hospitalization for HF in patients with an EF > 40% and NYHA (New York Heart Association) classes II-IV. Instead of having a significant effect on cardiovascular mortality, this outcome was mostly attributable to a 29% decreased likelihood of hospitalization for HF. The benefit was consistent across patients with or without diabetes [11].

Further, the outcomes of EMPAREG OUTCOME, CANVAS, and DECLARE-TIMI 58 trials were summarized in a meta-analysis. It was shown that while there was no difference in primary prevention [173], T2DM patients with atherosclerotic CVD experienced better cardiovascular outcomes. These results support the 2020 ESC guidelines’ suggestion that individuals with T2DM and established CVD obtain SGLT2 inhibitors as their first treatment [174]. Several additional or exploratory end points, such as those related to HF and renal disease, were also gathered from these trials.

In both a diuretic and a natriuretic context, a prolonged decrease in intravascular volume results in a decrease in preload and an improvement in the systolic and diastolic functions of the left ventricle [175]. This observation was made particularly in the abovementioned EMPA-TROPISM study, which demonstrated a substantial decrease in the end-systolic and diastolic volumes and a considerable rise in the LVEF (6% vs. 0.1%) when compared to a placebo.

Another hypothesis suggests that ketone bodies might prevent pathological remodeling. Class I histone deacetylases, which in HF can suppress pro-hypertrophic transcription [176,177,178], have been demonstrated to be inhibited by BHB in prior studies. On top of that, in models of ischemia or an elevated afterload, the overexpression of major ketone body oxidation enzymes such as BDH1 (D-beta-hydroxybutyrate dehydrogenase) guards against heart remodeling [179]. Moreover, in the SCOT knockout models, greater pathological remodeling is evidenced, suggesting some benefits of ketone bodies in cardiac remodeling [180].

Taken together, the increase in ketone body levels induced by SGLT2 inhibitors could potentially alleviate pathological remodeling and have a positive impact on the cardiac muscle. Nonetheless, additional research is necessary to elucidate this mechanism, which remains ambiguous.

The fundamental characteristics of clinical trials investigating the cardiovascular end points in individuals administered with the ketogenic agents, SGLT2 inhibitors, are succinctly outlined in Table 3.

### 4.4. Kidney Disease

Similar to the heart, the kidney is also a highly energetically and metabolically active organ. Hence, in cases of kidney failure and CKD, ketone bodies offer a promising oxygen-efficient energy source that can prevent kidney hypoxia [162,185]. Utilizing ketones as fuel reduces the risk of cell apoptosis, fibrosis, and the development of diabetic nephropathy, commonly known as diabetic kidney disease (DKD) [154]. SGLT2 inhibition induces ketogenesis, which then is believed to affect mTORC1 (the mammalian target of rapamycin complex 1) signaling. The inhibition of mTORC1 by ketones may subsequently prevent kidney damage, thus providing renoprotection [154,186].

Moreover, SGLT2 inhibitors activate the SIRT1 (sirtuin-1) gene regulator, which is responsible for gluconeogenesis and therefore ketogenesis. Certain genetic variations of SIRT1 have been linked to an increased susceptibility to developing diabetic nephropathy. It has specifically been suggested that the reno-protective effects of SGLT2 inhibitors may be due to their ability to stimulate SIRT1 genes [154,186,187].

Notably, the activation of SIRT-1, along with its associated effectors, PGC-1α (peroxisome proliferator-activated receptor gamma coactivator 1-alpha) and FGF21 (fibroblast growth factor 21), as well as the engagement of the SIRT1/AMPK (sirtuin 1/AMP-activated protein kinase) signaling pathway, is widely recognized as a central element contributing to the renoprotective attributes of SGLT2 inhibitors. While the precise underlying molecular mechanisms remain incompletely understood, a highly supported hypothesis suggests that the activation of the SIRT1/AMPK signaling pathway induces a state of energy deprivation. This metabolic transition is concomitant with reduced oxidative stress, the restoration of mitochondrial functionality, the mitigation of inflammatory processes, enhanced contractile activity, and an increased occurrence of autophagy. These coordinated cellular responses result from the widespread activation of SIRT-1 across all bodily cells as a consequence of SGLT2 inhibitor administration. This, in turn, leads to a systemic state of metabolic insufficiency, thereby strengthening the renoprotective benefits provided by these inhibitors [188,189].

Regarding kidney disease, three cardiovascular outcome trials in patients with T2DM, with or without prevalent CVD, showed that SGLT2 inhibitors might lower a composite of renal outcomes by 40–70%, including the requirement for dialysis and/or transplantation, the development of macroalbuminuria, the doubling of blood creatinine, and kidney mortality [8,163,182]. In regard to CKD end points, a CREDENCE (Canagliflozin and Renal Events in Diabetes with Established Nephropathy Clinical Evaluation) in patients with DKD and DAPA-CKD (Dapagliflozin and Prevention of Adverse Outcomes in CKD) in patients with any form of CKD, as well as other minor studies such as DELIGHT (Delay Of Impaired Glucose Tolerance By A Healthy Lifestyle Trial Abbreviation), DERIVE (A Study to Evaluate the Effect of Dapagliflozin on Blood Glucose Level and Renal Safety in Patients with T2DM), and DIAMOND (Effects of Dapagliflozin on Proteinuria in Non-Diabetic Patients With CKD), were designed in populations with or without diabetes at risk for or suffering from cardiac or renal pathologies [9,10,181,190,191,192,193,194,195,196].

The current ADA guidelines recommend canagliflozin, empagliflozin, or dapagliflozin in T2DM patients with DKD to slow the course of renal disease and cardiovascular events (estimated GFR of 25 mL/min/1.73 m^2^, and urinary albumin creatinine ≥ 300 mg/g) [197].

A compilation of clinical trials summarizing renal outcomes in patients treated with SGLT2 inhibitors is presented in Table 4.

### 4.5. Metabolic-Associated Fatty Liver Disease (MAFLD)

Metabolic-associated fatty liver disease (MAFLD) is the most prevalent liver disease globally, and more than 50% of people diagnosed with T2DM also have MAFLD. These two conditions are linked in a bidirectional pathological relationship, where MAFLD increases the likelihood of developing T2DM, while T2DM contributes to and accelerates the progression of MAFLD [198]. More than 30% of MAFLD patients develop NASH, which raises the risk of developing cirrhosis and HCC (hepatocellular cancer) [199,200,201]. The underlying pathophysiology of MAFLD has not yet been fully established despite its high frequency and possible clinical consequences, and there is no agreement on the typical diagnosis and course of action for either MALFD or NASH. Patients with both NASH and T2DM have compromised hepatic function as a result of chronic inflammation and the ensuing structural alterations brought on by hepatic fat buildup, which limits their options for anti-diabetic therapy [202]. In fact, TAG accumulates in the cytoplasm of hepatocytes (or hepatic cells), causing fatty liver disease, which then progresses to MAFLD and NASH [203].

The SGLT2 inhibitor, canagliflozin (CANA), induces significant transcriptional reprogramming and metabolic shifts, characterized by increased fatty acid oxidation, reduced hepatic steatosis, and elevated levels of the hepatokine FGF21. Interestingly, the induction of lipid oxidation and ketogenesis by CANA seems to proceed independently of FGF21, yet this hepatokine plays a critical role in the activation of lipolysis and adiposity reduction, illustrating a complex, dual pathway of metabolic regulation that mirrors a fasting-like metabolic paradigm [204].

When considered in the context of MAFLD, SGLT2 inhibitors’ ketogenic effect could provide therapeutic benefits. Insufficient hepatic ketogenesis, a major pathogenic mechanism implicated in MAFLD, influences the expression of genes associated with de novo lipogenesis, consequently leading to excess lipid accumulation in hepatocytes. Decreased ketone body levels can escalate mitochondrial stress and exacerbate triglyceride accumulation, expediting the progression of MAFLD to NASH through heightened oxidative stress [200,203,205,206].

Further adding to this potential therapeutic selection of SGLT2 inhibitors, evidence indicates that drugs like dapagliflozin or empagliflozin inhibit the activation of the NLR family pyrin domain-containing 3 (NLRP3) inflammasome. This mechanism is linked to NASH and, subsequently, to fibrogenesis. Hence, these inhibitors could potentially attenuate fibrosis progression and slow the transition of MAFLD to NASH. The control of metabolic regulation through both FGF21-dependent and independent pathways points towards the multifaceted capacities of SGLT2 inhibitors, highlighting their potential as comprehensive strategies for the management of metabolic conditions such as MAFLD and NASH [204,207,208].

In the context of the previously discussed mechanisms, SGLT2 inhibitors stimulate SIRT1 expression, thereby promoting gluconeogenesis and, in effect, amplifying ketogenesis. This process holds potential advantages not only for managing kidney dysfunction but also for MAFLD treatment. An elevated SIRT1 expression initiates an increase in fatty acid oxidation and a decrease in hepatic lipid storage by facilitating TAG catabolism. As such, SIRT1 activation emerges as a potential pathway for designing therapeutic strategies, potentially increasing the effectiveness of SGLT2 inhibitors in managing metabolic conditions. [154,187,209].

Importantly, animal studies have revealed encouraging evidence regarding the modulation of MAFLD/NASH through SGLT-2 inhibition [210,211]. For instance, in a mouse model exhibiting MAFLD-like symptoms, the administration of remogliflozin resulted in a substantial reduction in hepatic TAG levels and notable improvements in ALT (alanine transaminase) and AST (aspartate aminotransferase) levels [211]. These findings underscore not only the potential of SGLT2 inhibitors but also their capacity to complement strategies involving SIRT1 activation in addressing metabolic conditions.

Furthermore, in a murine model of NASH co-occurring with T2DM, dapagliflozin demonstrated a significant impact on glucose metabolism. This evidence suggests an effective reduction in ascites and an improvement in glycemic control in NASH-afflicted mice in comparison to their untreated counterparts [212].

Notwithstanding, further studies are required to determine the mechanisms by which SGLT-2 inhibitors influence steatohepatitis and fatty liver disease. Despite the ongoing inquiries, numerous trials involving individuals with T2DM have yielded compelling evidence supporting the potential of these medications to address both T2DM and MAFLD or NASH [120,213,214,215,216,217]. 

**Figure 4:**
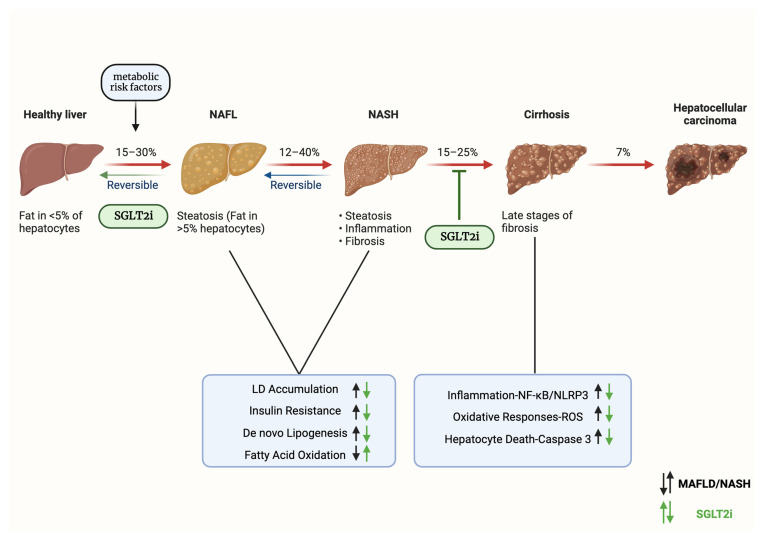
Clinical efficacy of SGLT2 inhibitors as ketogenic agents for metabolic-associated fatty liver disease treatment. Abbreviations: SGLT2i—sodium–glucose cotransporter 2 inhibitors; NAFL—nonalcoholic fatty liver; MAFLD—metabolic-associated fatty liver disease; NASH—nonalcoholic steatohepatitis; LD—lipid droplet; NF-κΒ—nuclear factor kappa B; NLRP3—NLR family pyrin domain containing 3; ROS—reactive oxygen species. Created with BioRender.com and accessed on 17 April 2021.

The therapeutic efficacy of SGLT2 inhibitors, particularly when employed as ketogenic agents in the management of MAFLD, is depicted in Figure 4.

## 5. Comparison of SGLT-2 Inhibitors with Other Anti-Diabetic Agents in Terms of Ketogenesis

One of the potential side effects of SGLT-2 inhibitors in terms of ketogenesis is an increased risk of DKA, a serious complication characterized by the accumulation of ketones in the blood [99,218,219]. Previously conducted randomized clinical trials have suggested the potential risk of DKA among patients treated with dapagliflozin. In the DECLARE-TIMI study, with a median duration of dapagliflozin exposure of 48 months, DKA was reported in 27 patients in the dapagliflozin (10 mg) group and 12 patients in the placebo group [163]. In the DAPA-HF study, DKA was reported in three patients with T2DM in the dapagliflozin group and no patients in the placebo group. Dapagliflozin was discontinued if DKA occurred due to safety concerns [9]. One example of the SmPCs (Summary of Product Characteristics) indicated that DKA associated with the use of dapagliflozin may be life-threatening and may be fatal (in rare cases). Patients are at particular risk if their food and fluid intake is rapidly reduced due to other medical conditions or procedures (e.g., following surgery). DKA can occur both immediately after starting therapy and even some time after initiating treatment with SGLT2 inhibitors. Clinicians should be alert for sudden vomiting, weakness, dehydration, and lethargy [220]. The occurrence of DKA in the past, associated with the use of SGLT-2 inhibitors, should be a contraindication to the re-use of this group of drugs [221].

Compared to other antidiabetic drugs, it is SGLT2 inhibitors that have the most clinically important potential for the development of DKA. Metformin does not increase the risk of DKA [222]. Other drugs, such as sulfonylureas, may lead to clinically significant hypoglycemia, followed by increased ketone production, mainly as a consequence of hypoglycemic compensatory mechanisms. Nonetheless, the risk of DKA is significantly lower in these cases than in the case of SGLT-2 inhibitors [223]. In the same way, DPP-4 (dipeptidyl peptidase-4) inhibitors, also known as gliptins, and GLP-1 receptor agonists do not significantly affect ketogenesis. Precisely, compared to DPP-4, SGLT2 inhibitors have a three-fold increased risk of DKA [224].

Contrarily, certain studies have yielded no significant disparity in the incidence of DKA across diverse antidiabetic medications. These findings underscore the importance of comprehensive evaluation and individualized treatment approaches, considering factors beyond the choice of specific antidiabetic agents [225,226].

A detailed comparison of SGLT-2 Inhibitors with other anti-diabetic drugs in terms of ketogenesis is provided in Table 5.

## 6. Future Directions Regarding the Ketogenic Effects of SGLT-2 Inhibitors

Many questions are still unanswered, despite the fact that SGLT2 inhibitors have changed the treatment of T2DM. First of all, the precise pathophysiological processes through which SGLT2 inhibitors can apply their benefits to the kidney and cardiovascular system are not entirely understood. Second, it is still unknown if these effects are isolated to particular populations with cardiac or renal illness or can be generalized to the entire diabetic population. However, as per D’Andrea et al., despite the initial concerns that employing SGLT2 inhibitors at elevated HbA1c levels might pose an increased risk, it is now established that individuals with T2DM can experience advantages from SGLT2 inhibitor usage irrespective of their glycemic control, and there is no heightened risk of adverse effects in those with increased HbA1c levels [227]. We have also observed the outcomes of trials that look at whether the positive effects of SGLT-2 inhibitors on HF and renal function can be sustained even in the absence of T2DM [9,10,193,228]. Specifically, SGLT2 inhibitors represent an effective treatment to reduce the progression of kidney disease or death from cardiovascular causes in patients with and without diabetes mellitus [229]. Another point to consider is that all of the SGLT2 inhibitors that have successfully completed a number of phase III and phase IV RCTs (randomized controlled trials) share the reduced need for HF hospitalization as well as the renal protective effects. Yet, there are some distinctions between them in terms of cardiovascular death, overall mortality, and negative consequences. According to the evidence, empagliflozin considerably decreased the incidence of cardiovascular death [152,168,175], but canagliflozin and dapagliflozin did not. Similar to empagliflozin and dapagliflozin, canagliflozin did not affect lower limb amputation incidence, whereas empagliflozin and dapagliflozin did [230]. It is challenging to compare these interventions directly because the study populations were different. Therefore, to determine if these results reflect a class effect or whether there is in fact a difference between them, head-to-head RCTs, well-planned observational studies, and real-world data are required. Then, when prescribing anti-diabetic medications, it is crucial to individualize the treatment to the specific patient’s needs. Additionally, glycemic control and renal function did not appear to be significantly affected by the increase in the SGLT2 inhibitors’ dosage, and the incidence of side effects did not rise.

One of the most important challenges of the future remains the impact of the use of concomitant medications on the risk of DKA. SGLT-2 inhibitors are used as monotherapy but also as another antidiabetic drug added, for example, to metformin, especially in the case of unsatisfactory glycemic control when using metformin alone. SGLT-2 inhibitors are also employed in patients exhibiting elevated cardiovascular risk, thereby presenting the possibility of interactions with other pharmacological agents employed in the realm of cardiovascular medicine. Another issue to consider is the additive effect of the drug combinations used together. Studies have shown that SGLT-2 inhibitors have a beneficial effect, for instance, on body weight, but whether other anti-diabetic agents can accelerate this effect remains unclear. As mentioned earlier, long-term studies are needed, for example, pragmatic clinical trials, to determine the long-term safety of using a combination of SGLT-2 inhibitors with inclusion/exclusion criteria as close as possible to routine clinical practice.

Another challenge is the use of SGLT2 inhibitors in patients diagnosed with T1DM. A 2021 meta-analysis including four randomized clinical trials (a total of 1691 patients) found that the use of SGLT2 inhibitors in this group of patients is not associated with a significantly higher risk of DKA than in the placebo group, at least in a relatively short follow-up of 24 weeks [231]. On the other hand, a retrospective cohort study showed a significantly higher risk of DKA in patients using insulin in combination with SGLT2 inhibitors [232]. Careful patient selection might be considered a potential mitigation strategy to minimize the risk of serious adverse reactions among T1DM patients [233]. Long-term studies can help evaluate the safety and efficacy of SGLT-2 inhibitors as a treatment option for patients diagnosed with T1DM.

Further research areas may also include aspects relevant from the point of view of basic science. Although the mechanism of action of SGLT-2 inhibitors is well understood, the mechanisms leading to DKA/ketoacidosis, also at a molecular level, remain insufficiently studied. A deeper understanding of the mechanisms could lead to the selection of patients who are known to be at a higher risk of developing DKA, even before the initiation of therapy. The development of biomarkers that can assist clinicians in making a therapeutic decision to include SGLT-2 inhibitors is highly desirable [234].

While understanding the fundamental mechanisms through which SGLT-2 inhibitors enhance ketogenesis remains crucial for the advancement of biomarker development, a more profound comprehension could potentially unveil novel targets for prospective anti-diabetic medication, consequently expanding the array of treatment alternatives available to patients. From a practical point of view, there is also a need to promote new molecules that improve metabolic parameters, that is, body weight or obesity, as well as renal protective effects [235].

## 7. Conclusions

In conclusion, SGLT-2 inhibitors are a promising class of medications for the treatment of T2DM and other metabolic diseases. Their unique mechanism of action, which is independent of insulin, makes them a valuable addition to the armamentarium of healthcare professionals. However, their use must be monitored, and patients have to be educated on both the benefits and potential risks of SGLT-2 inhibitors. Ongoing research will continue to shed light on the complex interplay between glucose metabolism, insulin sensitivity, and the gut microbiome, which may ultimately lead to more effective and personalized treatments for metabolic diseases.

Due to their complex and multidirectional mechanism of action, this originally antidiabetic group of drugs has been successfully used to treat patients with HF and patients with CKD as well [236,237]. Moreover, their therapeutic potential seems to be even wider than the indications studied so far.

Ongoing clinical trials evaluating the effects of SGLT-2 inhibitors, particularly canagliflozin, on cardiorenal outcomes in patients with DKD have shown promising results. These findings suggest that canagliflozin may emerge as an effective and safe therapy for improving outcomes in both diabetic and nondiabetic kidney disease [238]. As more data become available from these and other ongoing trials, the role of SGLT-2 inhibitors in the management of DKD is expected to become clearer and may lead to the incorporation of these drugs as a cornerstone of therapy for patients with this debilitating condition [96,97,101,238,239,240].

The synergism of SGLT-2 inhibitors and ketogenic diets represents a promising therapeutic approach for managing T2DM and obesity. By promoting weight loss and improving glycemic control, this combination may offer enhanced clinical benefits compared to either intervention alone. However, potential risks, such as the increased risk of DKA and AKI (acute kidney injury), must be carefully considered and monitored [66,96,97,98,101,240]. Further research is required to elucidate the optimal combination of these treatments and to develop strategies for mitigating their associated risks. Ultimately, an individualized approach to therapy, considering each patient’s unique clinical circumstances, will be essential in maximizing the therapeutic potential of SGLT-2 inhibitors and ketogenic diets in the management of T2DM and obesity.

Finally, with the incredibly high incidence of T2DM in the current population of emergency department patients, it is critical for clinicians to understand the possible complications of the treatment of this disease. SGLT-2 inhibitor medications are becoming very common encounters on patient medication lists, and clinicians should be aware of how these medications, alone or combined with dietary modifications, can result in significant pathology and even mortality if not appropriately treated [241].

## Figures and Tables

**Table 2:** The key features of SGLT-2 inhibitors and ketogenic diets, along with the potential advantages and disadvantages of their synergism.

	SGLT-2 Inhibitors	Ketogenic Diets
**Characteristics**	A class of drugs used to treat T2DM by blocking glucose reabsorption in the kidneys, which leads to increased urinary glucose excretion.	A low-carbohydrate, high-fat diet that forces the body to burn fat for energy instead of carbohydrates. This metabolic state is known as ketosis.
**Advantages of Synergy**	Improved glycemic control → both lower blood glucose levels, and their combined effect can lead to better glycemic control. Weight loss → a ketogenic diet can lead to significant weight loss, and SGLT-2 inhibitors have been shown to reduce body weight and body fat. Cardiovascular benefits → SGLT-2 inhibitors reduce the risk of cardiovascular events in people with type 2 diabetes, and a ketogenic diet may also improve cardiovascular health. Blood pressure reduction→ both have been shown to reduce BP. Lowered inflammation → a ketogenic diet may reduce inflammation in the body, and SGLT-2 inhibitors have been shown to have anti-inflammatory effects. Neuroprotective effects → a ketogenic diet has been shown to have neuroprotective effects, and SGLT-2 inhibitors may also have neuroprotective effects.	
**Disadvantages of Synergy**	Diabetic ketoacidosis → both SGLT-2 inhibitors and a ketogenic diet increase the risk of DKA in people with T1DM Dehydration → SGLT-2 inhibitors increase urinary glucose excretion, which can lead to dehydration, whereas a ketogenic diet can also lead to dehydration if not properly balanced with adequate fluid and electrolyte intake. Electrolyte imbalance → SGLT-2 inhibitors increase the urinary excretion of Na^+^ and K^+^, which can lead to electrolyte imbalances. A ketogenic diet can also affect electrolyte balance if not properly balanced with an adequate intake of Na^+^, K^+^, and Mg^2+^. Hypoglycemia → a ketogenic diet can lead to hypoglycemia in people with type 2 diabetes who are taking medications that lower blood glucose levels, such as insulin or sulfonylureas. SGLT-2 inhibitors can also increase the risk of hypoglycemia when used in combination with other glucose-lowering medications. Gastrointestinal symptoms → a ketogenic diet can cause gastrointestinal symptoms such as nausea, vomiting, and diarrhea. SGLT-2 inhibitors can cause gastrointestinal symptoms such as nausea and diarrhea. Genital infections → SGLT-2 inhibitors can increase the risk of genital infections such as yeast infections and UTIs. A ketogenic diet may also increase the risk of yeast infections due to the high-fat content.	

Abbreviations: SGLT-2—sodium–glucose cotransporter-2; T2DM—type 2 diabetes mellitus; BP—blood pressure; DKA—diabetic ketoacidosis; T1DM—type 1 diabetes mellitus; Na^+^—sodium cations; K^+^—potassium cations; Mg^2+^—magnesium cations; UTIs—urinary tract infections.

**Table 3:** Clinical trials investigating the cardiovascular outcomes of individuals treated with sodium–glucose cotransporter-2 inhibitors.

Clinical Trial	Type of Trial	Study Information	Duration	Outcomes
DECLARE-TIMI 58 [163]	Double-blind, placebo-controlled RCT (phase 3)	17,160 T2DM patients at high risk for CV events (only 7% of patients had an eGFR < 60 mL/min/1.73 m^2^) Dapagliflozin 10 mg vs. placebo	Up to 6 years	Cardiovascular death, nonfatal MI, nonfatal ischemic stroke; cardiovascular death, hospitalization because of HF Renal composite end point (≥40% decrease in eGFR to <60 and ESRD and renal or cardiovascular death, all-cause mortality)
DAPA-HF [9]	Double-blind, placebo-controlled RCT (phase 3)	4304 diabetic (68%) or non-diabetic patients with class II-IV HF Dapagliflozin 10 mg vs. placebo	36 months	Time to cardiovascular death or hospitalization for HF or an urgent HF visit Time to ≥50% sustained decline in eGFR or ESRD QoL score by questionnaire Time to death by any cause
DEFINE HF [181]	Double-blind, placebo-controlled RCT (phase 4)	263 diabetic or nondiabetic patients with class II and III HF Dapagliflozin 10 mg vs. placebo	12 weeks	Change in NTproBNP Change in SBP, weight, HbA1c, BNP, and QoL score by questionnaire
EMPA-REG OUTCOME [8]	Double-blind, placebo-controlled RCT (phase 3)	7020 T2DM patients at high risk for CV events and an eGFR ≥ 30 mL/min/1.73 m^2^ Empagliflozin 10 mg vs. Empagliflozin 25 mg vs. placebo	Up to 4.6 years	14% reduction in 3-point MACE (cardiovascular death, nonfatal MI, nonfatal stroke) pooled from 10 mg and 25 mg empagliflozin doses 35% reduction in hospitalization for HF, 39% reduction in the composite renal end point (new macroalbuminuria, doubling of serum creatinine and a GFR ≤ 45, renal replacement therapy, renal death)
EMPEROR-Reduced [10]	Double-blind, placebo-controlled RCT (phase 3)	3730 diabetic or non-diabetic patients with class II, III, or IV HF and an EF ≤ 40% Empagliflozin 10 mg vs. placebo (additionally to recommended treatment)	38 months	Cardiovascular death or adjudicated hospitalization for HF Change in eGFR Time to sustained reduction in eGFR Time to all-cause mortality Time to DM.
EMPEROR-Preserved [11]	Double-blind, placebo-controlled RCT (phase 3)	5988 diabetic or not diabetic patients with class II-IV HF and EF > 40% Empagliflozin 10 mg vs. placebo (in addition to usual therapy)	38 months	Cardiovascular death or adjudicated hospitalization for HF Change in eGFR Time to sustained reduction in eGFR Time to all-cause mortality Time to DM.
CANVAS [182]	Double-blind, placebo-controlled RCT (phase 3)	10,142 participants with T2DM and high CV risk Canagliflozin 100 mg (with an increase to 300 mg) vs. placebo	3.6 years	14% reduction in 3-point MACE (cardiovascular death, nonfatal MI, nonfatal stroke) 27% reduction in progression of albuminuria, 70% increase in regression of albuminuria, 40% reduction in the composite renal end point (40% reduction in eGFR, renal replacement therapy, renal death)
CREDENCE [183]	Double-blind, placebo-controlled RCT (phase 3)	3627 T2DM patients with stage 2 or 3 CKD and macroalbuminuria and on ACEIs/ARB (>30 y) Canagliflozin 100 mg daily vs placebo	4 years	ESRD, S-creatinine doubling, renal/cardiovascular death Cardiovascular death, nonfatal MI, nonfatal stroke, hospitalized UAP, hospitalized CHF, composite renal end point (ESRD, doubling of serum creatinine renal death)
VERTIS CV [184]	Double-blind, placebo-controlled RCT (phase 3)	8246 T2DM patients with established CV disease and an eGFR ≥ 30 mL/min/ 1.73 m^2^ Ertugliflozin 5 or 15 mg vs. placebo	Up to 6 years	Cardiovascular death, nonfatal MI, nonfatal stroke Cardiovascular death, nonfatal MI, nonfatal stroke and hospitalized UAP

Abbreviations: RCT—randomized controlled trial; CV—cardiovascular; eGFR—estimated glomerular filtration rate; MI—myocardial infarction; HF—heart failure; ESRD—end-stage renal disease/failure; QoL—quality of life; NTproBNP—N-terminal prohormone of brain natriuretic peptide; SBP—systolic blood pressure; HbA1c—hemoglobin A1c/glycohemoglobin; BNP—brain natriuretic peptide; GFR—glomerular filtration rate; EF—ejection fraction; DM—diabetes mellitus; ACEIs—angiotensin-converting enzyme inhibitors; ARBS—angiotensin receptor blockers; S-creatinine—serum creatinine; UAP—unstable angina pectoris; CHF—congestive heart failure; DECLARE-TIMI 58—dapagliflozin effect on cardiovascular events—thrombolysis in MI 58; DAPA-HF—dapagliflozin and prevention of adverse outcomes in HF; DEFINE-HF—dapagliflozin effects on biomarkers, symptoms and functional status in patients with HFrEF (heart failure with reduced ejection fraction); EMPA-REG OUTCOME—empagliflozin cardiovascular outcome event trial in T2DM; EMPEROR-Reduced—empagliflozin outcome trial in patients with chronic HFrEF; EMPEROR-Preserved—empagliflozin outcome trial in patients with chronic HFpEF (heart failure with preserved ejection fraction); CANVAS—canagliflozin cardiovascular assessment study; CREDENCE—canagliflozin and renal events in diabetes with established nephropathy clinical evaluation; VERTIS CV—evaluation of ertugliflozin efficacy and safety cardiovascular outcomes trial.

**Table 4:** Clinical trials evaluating the renal outcomes of individuals treated with the sodium–glucose cotransporter-2 inhibitors.

**Drug**	**Clinical Trial**	**Type of Trial**	**Study Information**	**Outcomes**
Dapagliflozin	DECLARE-TIMI 58 [163]	Double-blind, placebo-controlled RCT	7160 T2DM patients at high risk for CV events (only 7% with eGFR < 60 mL/min/1.73 m^2^) Dapagliflozin 10 mg vs. placebo	Composite of ≥40% reduction in eGFR, new ESRD, or death from renal or CV causes
	DAPA-CKD [193]	Double-blind, placebo-controlled RCT	4304 diabetic (68%) or non-diabetic patients suffering from CKD (UACR of 200–5000 mg/g and eGFR of 25–75 mL/min/1.73 m^2^) Dapagliflozin 10 mg vs. placebo	Composite of ≥50% sustained decline in eGFR or ESRD or CV or renal death Composite of CV death and hospitalization for heart failure Variation in albumin-to-creatinine ratio
	DELIGHT [194]	Placebo-controlled RCT	461 T2DM patients with albuminuria (UACR 30–3500 mg/g) and eGFR of 25–75 mL/min/1.73 m^2^, treated with ACEIs or ARBs Dapagliflozin 10 mg vs. Dapagliflozin 10 mg Saxagliptin 2.5 mg vs. placebo	
	DERIVE [195]	Double-blind, placebo-controlled RCT Randomized-double blind, cross-over trial	321 T2DM patients with CKD in stage 3A (eGFR of 45–59 mL/min/1.73 m^2^) Dapagliflozin 10 mg vs. placebo	Change from baseline in urine eGFR
	DIAMOND [196]		53 nondiabetic patients with CKD (24 h urinary protein excretion > 500 mg and ≤3500 mg, eGFR ≥ 25 mL/min/1.73 m^2^) on stable RAS blockade 27 received Dapagliflozin 10 mg then placebo, 26 received placebo then Dapagliflozin 10 mg	Mean proteinuria Measured GFR
Empagliflozin	EMPA-REG OUTCOME [8]	Double-blind, placebo-controlled RCT	7020 T2DM patients with high risk for CV events and eGFR ≥ 30 mL/min/1.73 m^2^ Empagliflozin 10 mg vs. placebo Empagliflozin 25 mg vs. placebo	Incident or worsening nephropathy Progression to macroalbuminuria Doubling of the serum creatinine level Initiation of renal-replacement therapy Post hoc composite of doubling of serum creatinine, renal replacement therapy, or death for renal causes Incident albuminuria
Canagliflozin	CANVAS [182] CREDENCE [190]	Double-blind, placebo-controlled RCT Double-blind, placebo-controlled RCT	5812 T2DM patients with high risk for CV events and eGFR > 30 mL/min/1.73 m^2^ Canagliflozin 100 or 300 mg vs. placebo 4401 T2DM patients with albuminuric CKD (eGFR of 30 to < 90 mL/min/1.73 m^2^) Canagliflozin 100 mg vs. placebo	Lower progression of albuminuria Composite of 40% reduction in eGFR, renal replacement therapy, or death from renal causes Composite of ESRD (dialysis, transplantation, or sustained eGFR < 15 mL/min/1.73 m^2^), Doubling of serum creatinine or death from renal or CV causes Composite of ESRD, a doubling of the creatinine level, or death from renal causes Composite of cardiovascular death, myocardial infarction, or stroke
Ertugliflozin	VERTIS CV [184]	Double-blind, placebo-controlled RCT	8246 T2DM patients with established CV disease and eGFR ≥ 30 mL/min/1.73 m^2^ Ertugliflozin 5 or 15 mg vs. placebo	Composite of death from renal causes, renal replacement therapy, or doubling of serum creatinine

Abbreviations: RCT—randomized controlled trial; T2DM—type 2 diabetes mellitus; CV—cardiovascular; eGFR—estimated glomerular filtration rate; ESRD—end-stage renal disease/failure; CKD—chronic kidney disease; UACR—urine albumin–creatinine ratio; ACEIs—angiotensin-converting enzyme inhibitors; ARBS—angiotensin receptor blockers; RAS—renin–angiotensin system; GFR—glomerular filtration rate; DECLARE-TIMI 58—dapagliflozin effect on cardiovascular events—thrombolysis in MI (myocardial infarction) 58; DAPA-CKD—dapagliflozin and prevention of adverse outcomes in CKD; DELIGHT—delay of impaired glucose tolerance by a healthy lifestyle trial; DERIVE—the effect of dapagliflozin on blood glucose level and renal safety in patients with T2DM; DIAMOND—effects of dapagliflozin on proteinuria in nondiabetic patients with CKD; EMPA-REG OUTCOME—empagliflozin cardiovascular outcome event trial in T2DM; CANVAS—canagliflozin cardiovascular assessment study; CREDENCE—canagliflozin and renal events in diabetes with established nephropathy clinical evaluation; VERTIS CV—evaluation of ertugliflozin efficacy and safety cardiovascular outcomes trial.

**Table 5:** Comparison of SGLT-2 inhibitors with other anti-diabetic drugs with respect to ketogenesis.

Anti-Diabetic Agent	Mechanism of Action	Ketogenic Potential
SGLT-2 Inhibitors	Inhibit glucose reabsorption in the kidney, leading to increased urinary glucose excretion	Promote mild ketogenesis due to increased free fatty acid availability and decreased insulin secretion
Metformin	Decreases hepatic glucose production and improves insulin sensitivity	Low potential → Does not promote ketogenesis
Sulfonylureas	Stimulate insulin secretion from β cells in the pancreas	Low potential → Does not promote ketogenesis
DPP-4 Inhibitors	Inhibit the enzyme DPP-4, which breaks down incretin hormones that stimulate insulin secretion	Low potential → Does not promote ketogenesis
GLP-1 Receptor Agonists	Mimic the action of GLP-1, an incretin hormone that stimulates insulin secretion and decreases glucagon secretion	Low potential → Does not promote ketogenesis
Insulin	Facilitates glucose uptake by cells and decreases hepatic glucose production	Low potential → Does not promote ketogenesis

Abbreviations: SGLT-2—sodium–glucose cotransporter-2; β—beta; DPP-4—dipeptidyl peptidase 4 (gliptins); GLP-1—glucagon-like peptide-1.

## Data Availability

Not applicable.
